# Child abuse and pubertal timing: what is the role of child sex and identity of the perpetrator?

**DOI:** 10.1186/s12888-024-05683-6

**Published:** 2024-04-01

**Authors:** V. Steger, S. Stadelmann, L. White, M. Döhnert

**Affiliations:** 1 LIFE – Leipzig Research Centre for Civilization Diseases, University of Leipzig, Philipp-Rosenthal-Straße 27, 04103 Leipzig, Germany; 2 Department of Child and Adolescent Psychiatry, Psychotherapy and Psychosomatic Medicine, University of Leipzig, Liebigstraße 20, 04103 Leipzig, Germany; 3 Department of Child and Adolescent Psychiatry, Psychotherapy and Psychosomatic Medicine, St Elisabeth & St Barbara Hospital, Barbarastraße 4, 06110 Halle (Saale), Germany; 4 Bremen, Germany

**Keywords:** Child abuse, Child neglect, Child physical abuse, Child emotional abuse, Early puberty, Menarche, Sex, Parental perpetrator, Socioeconomic status, Body-Mass-Index

## Abstract

**Background:**

This study investigated the association between child abuse [child neglect (CN), emotional (CEA) and physical abuse (CPA)] and early puberty with special regard to sex-specific effects concerning child and parental perpetrator*.*

**Methods:**

Data assessment took place within the framework of the LIFE Child Depression study, a longitudinal study on the development of depressive symptoms and disorders between child- and adulthood in Leipzig, Germany. A sample of 709 children (8–14 years) was recruited from the general population and via psychiatric hospitals. Data on pubertal status were assessed using an instrument for self-assessment of tanner stages (scales of physical pubertal development). Information on menarche was provided by parents. The Parent–Child Conflict Tactics Scales (CTS-PC) served for data on child abuse.

**Results:**

Regarding physical puberty markers, significant correlations were found, especially with child neglect (CN) and child emotional abuse (CEA). Regression analyses, controlling for Body-Mass-Index (BMI) and Socioeconomic Status (SES), revealed that children affected by child neglect perpetrated by mother (CN_m_) and child emotional abuse (CEA) parent-non-specifically enter puberty significantly earlier. Sex-specific analyses identified child neglect perpetrated by mother (CN_m_) to be associated with early puberty in girls and child emotional abuse perpetrated by father (CEA_f_) with early puberty in boys. Concerning the onset of menstruation, there was a significant positive correlation between early menarche and parent-specific and non-specific child neglect (CN), as well as between early menarche and child emotional abuse perpetrated by the mother (CEA_m_). In regression models that controlled for Body-Mass-Index (BMI) and Socioeconomic Status (SES) no significant associations were maintained. Child physical abuse (CPA) was not associated with early puberty.

**Conclusion:**

Results outlined child neglect (CN) and child emotional abuse (CEA) to be sex- and perpetrator-specific risk factors for early pubertal development. Knowledge of sex- and perpetrator-specific effects could help clinicians to specify their diagnostic process and to define differential prevention and treatment goals for children with experiences of CN and CEA. Further research on the sex-specific impact of parental CN and CEA on girls’ and boys’ puberty is needed.

**Supplementary Information:**

The online version contains supplementary material available at 10.1186/s12888-024-05683-6.

## Background

The past decades have witnessed a growing interest in child abuse and its sequelae. According to the World Health Organisation child abuse includes forms of child sexual (CSA), physical (CPA), emotional abuse (CEA), child neglect (CN), as well as “exploitation, which results in actual or potential harm to the child’s health, survival, development or dignity in the context of a relationship of responsibility, trust or power” [1]. Kang et al. [2] and Metzler et al. [3] describe children’s health to be susceptible to adverse childhood experiences (ACE), including child abuse, leaving them at risk of disrupted neurodevelopment, social/emotional/cognitive impairment, adoption of health risk behaviour and shorter lifespans. Changes in hormonal processes due to ACE have been identified by Heim et al. [4]; Jaffee et al. [5] and White et al. [6]. White et al. [6] described that early life stress may initially lead to an increase of cortisol. Once stress has become chronic cortisol levels drop. Moreover, they revealed an association between low concentrations of cortisol and advanced pubertal state. In line with these results, Wingfield, Sapolsky et al. [7] examined the interaction between hypothalamic–pituitary–adrenal (HPA) and hypothalamic–pituitary–gonadal (HPG) axis showing sexual hormones to be under the influence of cortisol levels. Increased levels of cortisol in reaction to high stress may lead to an increase of the sexual hormones luteinizing hormone (LH) and follicle stimulating hormone (FSH). In 2009 Ellis et al. [8] already postulated pubertal timing to be influenced by early life stress following child abuse. Existing literature stresses that shifts in pubertal timing may potentially carry important repercussions for physical and mental health [9]. Early pubertal timing, for example, has shown to be associated with increased depressive symptoms [10], active asthma (especially in male children), the risk of incident asthma (in both sexes) [11], a higher adult BMI, as well as a greater risk of obesity [12]. Concerning both early and delayed puberty, recent studies suggest a negative effect for cardiovascular health [13].

Depending on the subtype of child abuse, pubertal timing seems to be affected differently. CSA has been repeatedly shown to be associated with early menarche [14, 15]. Life history theories claim that being raised in harsh environments may predispose to an early pubertal onset, reflecting an overarching selective advantage of faster maturation (so-called rapid life history strategies) in hostile environments, for example, via a younger reproductive age [16]. Through inflicting potential costs at the individual level (e.g., fewer available resources for childcare), early reproduction, in turn raises the chances for a species to survive, placing a premium on the quantity (not the quality) of offspring [17].

Despite the appeal of this conceptual model, studies have yielded contradictory patterns. Thus, Li et al. [18] observed CSA to be linked to both early and late pubertal onset, while CN was only linked to later onset. CPA and CEA were associated with late menarche and late gonadal hair growth in boys. CEA and its effects on puberty have generally only been poorly understood. In their analyses on child abuse and pubertal timing Negriff et al. [19] identified significant sex differences. Boys with CN were more likely to show a later onset of puberty. Once entered, puberty progressed more quickly. In line with established empirical patterns, CSA in girls was associated with an early pubertal onset, but slow pubertal development. In conclusion, pubertal timing not only appears to be under the influence of the subtype of child maltreatment, but also seems to vary as a function of sex.

Ample theory and data suggest that sex differences can arise in different emotional and hormonal responses to stress. The Adaptive Calibration Model by Del Giudice et al. [20], outlines sex differences in stress responsivity, specifying patterns of more female-typical vigilant responsivity (high responsivity in dangerous/unpredictable environments) and more male-typical unemotional patterns (low responsivity following severe/traumatic stress). In experimental studies on rats Bangasser et al. [21] identified sex differences in the corticotropin releasing factor (CRF) receptor and showed females to be predisposed to greater stress-related anxiety, more sensitive to low levels of CRF and less adaptable to high levels. Moreover, sex differences concerning stress regulation and sexual maturation contribute to variances in pubertal timing among males and females [19, 22]. As research on the association between early puberty following child abuse and the role of sex is very limited, we want to focuso sex-specific aspects.

In general, prevalence rates of child abuse seem to vary in respect of both the child’s and the perpetrator’s sex [23, 24]. Due to historic shifts in parenting styles, differences between parenting of boys versus girls still exist but have become quite small [23]. Concerning perpetration, abusive behaviour is most commonly (in about half the cases) exhibited by both parents [25]. Nevertheless, different behaviours and characteristics among males and females are being reflected in different parenting strategies [26]. Along with the observation that parents spend more time with same-sex children than with opposite-sex children, Endendijk et al. [27] showed that children and adolescents tend to especially imitate the behaviour of their same-sex parent, such as parental smoking and drinking behaviour. In line with these thoughts, Oshio and Umeda [28] observed a sex-specific impact of parental childhood abuse on children’s problem behaviour with strong mother–daughter and father–son linkages.

To the best of our knowledge, analyses on early puberty following child abuse and the role of the identity or familial relationship to the individual who perpetrated child maltreatment have not been conducted to date. As puberty is a time in which adolescents undergo a sensitive process of self-reflection and identity searching [29], we assume male and female pubertal timing to be affected differently by sex-specific parenting and relationship processes. In order to fill in on existing research we additionally explored parent-specific perpetrator effects.

The body mass index (BMI) and the socioeconomic status (SES) have been identified as common mediating factors for early pubertal maturation. James-Todd et al. [30] found lower SES at 7 years and reductions in SES in early childhood both to be associated with an earlier age at menarche. Deardorff et al. [31] demonstrated similar results among Black and Hispanic girls in comparison to white girls. Concerning the BMI both Kaplowitz et al. [32] and Hoyt et al. [33] displayed associations between early puberty and higher BMI. As a consequence, we considered both parameters in our analyses.

This study aimed to analyse whether child abuse is associated with pubertal timing. Focussing on a child sample (participants aged 8–14 years), we firstly hypothesized that child abuse is associated with early puberty depending on the subtype of child abuse (CN, CEA or CPA). Secondly, we hypothesized variances in results in respect of the parental perpetrator’s and thirdly the participant’s sex. As high body-mass-index (BMI) and low socioeconomic status (SES) have been shown to be associated with early puberty [31, 33], we included both mediating factors in our analyses. Concerning our fourth hypothesis, we expected both high BMI and low SES to be correlated with early pubertal onset.

## Methods

### Study design and participants

Data were collected in an urban area in the eastern part of Germany (population of approx. 1 million) within the framework of the LIFE Child Depression Study. The longitudinal study is part of LIFE—Leipzig Research Centre for Civilization Diseases at the University of Leipzig. A community sample and a clinical sample from two local child psychiatric in- and outpatient services were recruited to include children with a wide variety in psychiatric symptoms and family backgrounds (for details see [34]). The sample of the present study encompasses n = 709 children (boys = 358 (50.5%), girls = 351 (49.5%), mean age: 11.4 years, SD: 1.94).^1^

Inclusion criteria were pre- and early adolescents aged 8–14 years and fluency in German. To ensure sufficient text comprehension, an IQ < 80 was an exclusion criterion. Participants diagnosed with autism or current psychotic disorder were also excluded to decrease the impact of confounding variables. We used data of children and parents of the first assessment wave on children’s biological development and child abuse. To complete data on age at menarche we also took data from first follow-up assessment (participants aged 10–16 years) (see Table 1). Our study protocol was approved by the Ethics Committee of the University of Leipzig. Parents or custodian gave written informed consent to contribute.

**Table 1:** Sample descriptives

	All	Female	Male
*N* (%)	709	351 (49.5)	358 (50.5)
Age mean (*SD*)	11.4 (1.94)	11.43 (1.97)	11.37 (1.92)
BMI mean (*SD*)	18.75 (3.55)	18.77 (3.52)	18.72 (3.59)
SES			
Low *N* (%)	159 (23.6)	75 (22.2)	84 (25.0)
Middle *N* (%)	288 (42.7)	147 (43.5)	141 (42.0)
High *N* (%)	227 (33.7)	116 (34.3)	111 (33.0)
Menarche mean age (*SD*)	(–)	12.15 (1.15)	(–)

*BMI* Body-Mass-Index, *SES* socioeconomic status, *SD* standard deviation

### Measures

#### Child abuse

To assess data on child abuse, children completed the Parent–Child Conflict Tactics Scales (CTS-PC). The CTS-PC [35] is a screening instrument to ascertain presence and degree of child abuse by parents, such as CN (including emotional and physical neglect), CPA and CEA, within the past year. Items concerning non-violent disciplines and open questions were excluded. Every item is listed twice distinguishing in-between the perpetrator (mother/father) of child abuse (Additional file 1). For analyses, we calculated the arithmetic mean of all assigned items for the three subtypes of child abuse and dichotomized the sum score of each child abuse subtype by mean split. For means see Table 2. For each we formed a parent-non-specific and a parent-specific variable.

**Table 2:** Sample prevalence of child abuse

Type of child abuse	All	Female	Male	Test (*p*)
	*N* (total)	*N*	*N*	
Child neglect (CN)				
CN (*N*)	659	335	324	χ^2^ = 0.01, *p* = 0.932, Cramer’s V = 0
Mean (*SD*)	0.36 (0.48)	0.36 (0.48)	0.36 (0.48)	
Yes (%)	237 (36.0)	121 (36.1)	116 (35.8)	
No (%)	422 (64)	214 (63.9)	208 (64.3)	
CN_m_ (*N*)	659	334	325	χ^2^ = 0.71, *p* = 0.399, Cramer’s V = 0.03
Mean (*SD*)	0.39 (0.49)	0.4 (0.49)	0.37 (0.48)	
Yes (%)	254 (38.5)	134 (40.1)	120 (36.9)	
No (%)	405 (61.5)	200 (59.9)	205 (63.1)	
CN_f_ (*N*)	587	300	287	χ^2^ = 0.01, *p* = 0.946, Cramer’s V = 0
Mean (*SD*)	0.33 (0.47)	0.33 (0.47)	0.32 (0.47)	
Yes (%)	191 (32.5)	98 (32.7)	93 (32.4)	
No (%)	396 (67.5)	202 (67.3)	194 (67.6)	
Child physical abuse (CPA)				
CPA (*N*)	647	330	317	χ^2^ = 4.67, *p* = 0.031, Cramer’s V = 0.09
Mean (*SD*)	0.28 (0.45)	0.24 (0.43)	0.32 (0.47)	
Yes (%)	179 (27.7)	79 (23.9)	100 (31.5)	
No (%)	468 (72.3)	251 (76.1)	217 (68.5)	
CPA_m_ (*N*)	659	334	325	χ^2^ = 1.34, *p* = 0.247, Cramer’s V = 0.05
Mean (*SD*)	0.32 (.47)	0.3 (0.46)	0.35 (0.48)	
Yes (%)	213 (32.3)	101 (30.2)	112 (34.5)	
No (%)	446 (67.7)	233 (69.8)	213 (65.5)	
CPA_f_ (*N*)	587	300	287	χ^2^ = 4.45, *p* = 0.035, Cramer’s V = 0.09
Mean (*SD*)	0.26 (0.44)	0.22 (0.42)	0.3 (0.46)	
Yes (%)	151 (25.7)	66 (22.0)	85 (29.6)	
No (%)	436 (74.3)	234 (78.0)	202 (70.4)	
Child emotional abuse (CEA)				
CEA (*N*)	657	333	324	χ^2^ = 2.69, *p* = 0.101, Cramer’s V = .06
Mean (*SD*)	0.4 (0.49)	0.37 (0.48)	0.43 (0.5)	
Yes (%)	263 (40.0)	123 (36.9)	140 (43.2)	
No (%)	394 (60.0)	210 (63.1)	184 (56.8)	
CEA_m_ (*N*)	659	334	325	χ^2^ = 1.97, *p* = 0.161, Cramer’s V = 0.06
Mean (*SD*)	0.34 (0.48)	0.32 (0.47)	0.37 (0.48)	
Yes (%)	226 (34.3)	106 (31.7)	120 (36.9)	
No (%)	433 (65.7)	228 (68.3)	205 (63.1)	
CPA_f_ (*N*)	587	300	287	χ^2^ = 0.02, *p* = 0.877, Cramer’s V = 0.01
Mean (*SD*)	0.4 (0.49)	0.4 (0.49)	0.39 (0.49)	
Yes (%)	233 (39.7)	120 (40.0)	113 (39.4)	
No (%)	354 (60.3)	180 (60.0)	174 (60.6)	

*CN*_*m/f*_ child neglect perpetrated by mother/father, *CPA*_*m/f*_ child physical abuse perpetrated by mother/father, *CEA*_*m/f*_ child emotional abuse perpetrated by mother/father, *SD* standard deviation

#### Pubertal timing

To assess pubertal status, we used a self-rating questionnaire on puberty related physical characteristics by Morris [36]. It included sex-specific drawings depicting five stages of pubertal development (thelarche/gonadarche and pubarche) according to Tanner (0 = B/PH1, 1 = B/PH2, 2 = B/PH3, 3 = B/PH4, 4 = PH5). Stages were described in an accompanying text below. Taking all ratings into account, both data on thelarche/gonadarche and pubarche were evaluated and assigned to one out of five Tanner stages (0 = tan1, 1 = tan2, 2 = tan3, 3 = tan4, 4 = tan5). Since participants were at different ages at moment of data assessment, we calculated each participant’s standardized residual value expressing pubertal status corrected for age (**SR-Pub**). Standardized residuals are known as measure of strength of the difference between observed and expected values. The new parameter **SR-Pub** expressed the degree a child diverges from the value on tanner scale that would be expected by child abuse in respect of child abuses chronological age. A more positive value identified an earlier, a more negative value a later pubertal timing.

#### Menarche

As part of an anamnestic questionnaire completed by parents, age at daughters’ menarche was captured. Data were collected twice at t1 and t2, since not every girl had reached menarche at t1. Thereby, we focused on data at t1 and completed missing data at t1 with answers given at t2. In line with analyses concerning pubertal status, we used binary regression analyses to calculate each girl’s standardized residual value (**SR-Men**). The parameter **SR-Men** expressed the degree menarche diverges from expected age of menarche according to their biological age. A more positive value identified an earlier, a more negative value a later menarche.

#### Body-Mass-Index (BMI)

As part of the physical examination, we collected data on children’s height and weight, using them to calculate the BMI [weight (kg)/height(m^2^)]. See Table 1

#### Socioeconomic status (SES)

The SES was assessed using a multidimensional index score, encompassing education and occupational qualification, occupational status and net income [37]. Referring to cut-offs, we classified SES into low, intermediate and high. SES of families was based on the highest score among the two parents at the time data was collected. See Table 1. For baseline characteristics concerning child abuse and socioeconomic status (SES) see Table 3.

**Table 3:** Baseline characteristics concerning child abuse and socioeconomic status (SES)

	*N* (in total)	SES			Test (*p*)
		Low	Med	High	
Child neglect (CN)					
All (%)	630 (100)	132 (21.0)	276 (43.8)	222 (35.2)	χ^2^ = 16.8, *p* = 0.001, Cramer’s V = 0.16 CNyes: SESlow > SEShigh
Yes (%) (*SR*)	226	63 (27.9) (2.3)	104 (46.0) (0.5)	59 (26.1) (− 2.3)	
No (%) (*SR*)	404	69 (17.1) (-1.7)	172 (42.6) (-0.4)	163 (40.3) (1.7)	
CN_m_	630 (100)	132 (21.0)	276 (43.8)	222 (35.2)	χ^2^ = 18.65, *p* = 0.001, Cramer’s V = 0.17 CNyes: SESlow > SEShigh
Yes (%) (*SR*)	243	69 (28.4) (2.5)	109 (44.9) (.2)	65 (26.7) (− 2.2)	
No (%) (*SR*)	387	63 (16.3) (− 2)	167 (43.2) (− 0.2)	157 (40.6) (1.8)	
CN_f_	564 (100)	95 (16.8)	254 (45.0)	215 (38.1)	χ^2^ = 9.66, *p* = .008, Cramer’s V = 0.13
Yes (%) (*SR*)	183	36 (19.7) (0.9)	94 (51.4) (1.3)	53 (29.0) (− 2)	
No (%) (*SR*)	381	59 (15.5) (− 0.6)	160 (42.0) (− 0.9)	162 (42.5) (1.4)	
Child physical abuse (CPA)					
All (%)	619 (100)	127 (20.5)	272 (43.9)	220 (35.5)	χ^2^ = 5.89, *p* = .053, Cramer’s V = 0.1
Yes (%) (*SR*)	172	46 (26.7) (1.8)	72 (41.9) (− 0.4)	54 (31.4) (− 0.9)	
No (%) (*SR*)	447	81 (18.1) (− 1.1)	200 (44.7) (0.3)	166 (37.1) (0.6)	
CPA_m_	630 (100)	132 (21.0)	276 (43.8)	222 (35.2)	χ^2^ = 8.52, *p* = 0.014, Cramer’s V = 0.12
Yes (%) (*SR*)	205	56 (27.3) (2)	88 (42.9) (− 0.2)	61 (29.8) (− 1.3)	
No (%) (*SR*)	425	76 (17.9) (− 1.4)	188 (44.2) (0.1)	161 (37.9) (0.9)	
CPA_f_	564 (100)	95 (16.8)	254 (45.0)	215 (38.1)	χ^2^ = 0.33, *p* = 0.848, Cramer’s V = 0.02
Yes (%) (*SR*)	147	27 (18.4) (− 0.3)	65 (44.2) (0.1)	55 (37.4) (0.1)	
No (%) (*SR*)	417	68 (16.3) (− 0.3)	189 (45.3) (0.1)	160 (38.4) (0.1)	
Child emotional abuse (CEA)					
All (%)	628 (100)	131 (20.9)	276 (43.9)	221 (35.2)	χ^2^ = 4.93, *p* = .085, Cramer’s V = 0.09
Yes (%) (*SR*)	250	60 (24.0) (1.1)	114 (45.6) (0.4)	76 (30.4) (− 1.3)	
No (%) (*SR*)	378	71 (18.8) (− 0.9)	162 (42.9) (− 0.3)	145 (38.4) (1)	
CEA_m_	630 (100)	132 (21.0)	276 (43.8)	222 (35.2)	χ^2^ = 7.76, *p* = 0.021, Cramer’s V = 0.11
Yes (%) (*SR*)	211	54 (25.6) (1.5)	97 (46.0) (0.5)	60 (28.4) (− 1.7)	
No (%) (*SR*)	419	78 (18.6) (− 1)	179 (42.7) (− 0.3)	162 (38.7) (1.2)	
CEA_f_	564 (100)	95 (16.8)	254 (45.0)	215 (38.1)	χ^2^ = 1.59, *p* = 0.453, Cramer’s V = 0.05
Yes (%) (*SR*)	225	43 (19.1) (0.8)	101 (44.9) (0)	81 (36.0) (− 0.5)	
No (%) (SR)	339	52 (15.3) (− 0.7)	153 (45.1) (0)	134 (39.5) (0.4)	

Post hoc analyses are based on the standardized residual method

*CN*_*m/f*_ child neglect perpetrated by mother/father, *CPA*_*m/f*_ child physical abuse perpetrated by mother/father, *CEA*_*m/f*_ child emotional abuse perpetrated by mother/father, *SR* standardized residuum, *χ*^*2*^ Chi-square

#### Statistical analysis

To analyse data, we used the statistical software IBM SPSS Statistics 22. Bivariate associations of CN, CEA and CPA with SR-Pub/Men were estimated using correlation analyses. Calculations were conducted for each subtype of child abuse including parent-non-specific- and parent-specific data. To explore sex-specific effects of child abuse on puberty, we conducted sex-specific correlation analyses.

To test our first hypothesis, we ran regression analyses on the effect of child abuse (CN, CEA and CPA) on pubertal timing (regarding both physical pubertal markers and onset of menstruation) controlling for BMI and SES (according to our fourth hypothesis). First, the parent-non-specific effects of all subtypes of child abuse were analysed. Second, we examined the parent-specific impact of each subtype of child abuse (CN/CEA/CPA perpetrated by mother/father [CN/CEA/CPA_(m/f)_]) to test our second hypothesis. To answer our third hypothesis concerning sex effects among participants, all regression analyses were run sex-non- and specifically.

## Results

### Child abuse and SR-Pub

#### Correlation analyses

There was a significantly positive correlation between SR-Pub and CN and CEA parent-non-specifically. In sex-dependent and parent-specific analyses, CN_m_ among girls was positively associated with SR-Pub. We also observed a significantly positive correlation between SR-Pub and CEA_f_ among boys and CEA_m_ among girls. CPA did not correlate with SR-Pub. BMI was significantly positive associated with CN and CEA, especially among girls. A significantly negative correlation was found between BMI and CPA in boys. For further information see Additional file 2: bivariate associations/correlation analyses.

#### Regression analyses

In parent- and sex-non-specific calculations, controlling for BMI and SES, CEA showed a significantly positive main effect on SR-Pub. In contrast, CN and CPA were not found to be associated with SR-Pub.

Parent-specific analyses revealed CN_m_ and CEA_f_ to have a significantly positive effect on SR-Pub. CPA was not found to be associated with SR-Pub. BMI showed a significantly positive effect on SR-Pub throughout all calculations.

Concerning regression analyses on boys, CEA_f_ appeared to be a significant predictor for early puberty. Concerning CN and CPA, as well as BMI no significant effects were found.

Regarding regression analyses on girls, CN_m_ was significantly associated with SR-Pub. CEA and CPA had no significant effect on SR-Pub. BMI showed a significantly positive effect on SR-Pub throughout all calculations.

SES had no significant effect on SR-Pub, neither in parent- and sex-specific nor non-specific calculations, which is why we excluded results in table. See Table 4

**Table 4:** Regression analyses SR-Pub—child abuse

Gender-independent					Male					Female				
Predictor	β	*B (SE)*	95% CI for *B*		Predictor	β	*B (SE)*	95% CI for *B*		Predictor	β	*B (SE)*	95% CI for *B*	
			Lower	Upper				Lower	Upper				Lower	Upper
Child abuse perpetrator-independent														
Step1					Step1					Step1				
CN	0.07	0.09	− 0.02	0.32	CN	0.05	0.13	− 0.15	0.38	CN	0.09	0.11	− 0.05	0.40
CPA	− 0.03	0.10	− 0.26	0.12	CPA	− 0.02	0.14	− 0.33	0.23	CPA	− 0.04	0.14	− 0.35	0.19
CEA	0.15***	0.09	0.13	0.49	CEA	0.18**	0.14	0.12	0.66	CEA	0.12	0.12	− 0.01	0.47
Step2					Step2					Step2				
CN	0.07	0.09	− 0.04	0.30	CN	0.06	0.14	− 0.14	0.39	CN	0.06	0.11	− 0.11	0.33
CPA	− 0.02	0.10	− 0.24	0.14	CPA	− 0.02	0.14	− 0.32	0.25	CPA	− 0.04	0.13	− 0.35	0.18
CEA	0.14**	0.09	0.10	0.46	CEA	0.18**	0.14	0.11	0.65	CEA	0.10	0.12	− 0.05	0.42
BMI	0.14***	0.01	0.02	0.06	BMI	0.06	0.02	− 0.02	0.05	BMI	0.24***	0.02	0.03	0.09
Child neglect (CN) perpetrator-dependent														
Step1					Step1					Step1				
CN_m_	0.13*	0.11	0.04	0.47	CN_m_	0.11	0.19	− 0.12	0.61	CN_m_	0.14	0.13	0	0.52
CN_f_	− 0.04	0.11	− 0.30	0.15	CNf	− 0.05	0.19	− 0.48	0.27	CN_f_	− 0.02	0.14	− 0.32	0.23
Step2					Step2					Step2				
CN_m_	0.13*	0.11	0.06	0.48	CN_m_	0.12	0.19	− 0.12	0.62	CN_m_	0.14*	0.13	0.02	0.52
CN_f_	− 0.05	0.11	− 0.31	0.12	CN_f_	− 0.05	0.19	− 0.48	0.27	CN_f_	− 0.07	0.14	− 0.41	0.12
BMI	0.15***	0.01	0.02	0.07	BMI	0.02	0.02	− 0.03	0.04	BMI	0.28***	0.02	0.05	0.11
Child physical abuse (CPA) perpetrator-dependent														
Step1					Step1					Step1				
CPA_m_	− 0.01	0.10	− 0.21	0.19	CPA_m_	− 0.02	0.15	− 0.35	0.25	CPA_m_	0.01	0.14	− 0.24	0.29
CPA_f_	0.06	0.11	− 0.07	0.36	CPA_f_	0.11	0.16	− 0.06	0.57	CPA_f_	0.02	0.15	− 0.26	0.33
Step2					Step2					Step2				
CPA_m_	− 0.01	0.10	− 0.21	0.18	CPA_m_	− 0.02	0.15	− 0.35	0.25	CPA_m_	0.02	0.13	− 0.21	0.30
CPA_f_	0.06	0.11	− 0.08	0.35	CPA_f_	0.11	0.16	− 0.06	0.57	CPA_f_	− 0.01	0.15	− 0.32	0.26
BMI	0.14***	0.01	0.02	0.06	BMI	0.02	0.02	− 0.03	0.04	BMI	0.28***	0.02	0.04	0.11
Child emotional abuse (CEA) perpetrator-dependent														
Step1					Step1					Step1				
CEA_m_	0.05	0.11	− 0.10	0.32	CEA_m_	− 0.04	0.16	− 0.39	0.24	CEA_m_	0.14*	0.14	0.01	0.57
CEA_f_	0.10*	0.10	0.01	0.41	CEA_f_	0.20**	0.16	0.12	0.74	CEA_f_	0	0.13	− 0.27	0.26
Step2					Step2					Step2				
CEA_m_	0.05	0.11	− 0.11	0.30	CEA_m_	− 0.04	0.16	− 0.39	0.24	CEA_m_	0.11	0.14	− 0.03	0.51
CEA_f_	0.09	0.10	− 0.02	0.39	CEA_f_	0.20**	0.16	0.12	0.74	CEA_f_	− 0.03	0.13	− 0.31	0.20
BMI	0.13**	0.01	0.01	0.06	BMI	0	0.02	− 0.04	0.04	BMI	0.26***	0.16	0.04	0.10

When including the SES in the regression analyses the results basically remained the same

*CN*_*m/f*_ child neglect perpetrated by mother/father, *CPA*_*m/f*_ child physical abuse perpetrated by mother/father, *CEA*_*m/f*_ child emotional abuse perpetrated by mother/father, *BMI* Body-Mass-Index, *β* standardized beta value, *B(SE)* standard errors of *B*, *95% CI for B* 95% confidence interval for *odds ratio*

Child abuse perpetrator/gender-independent: Step 1: *R*^2^ = 0.03, *p* = 0.001; Step 2: *R*^2^ = 0.05, *p* < 0.001; child abuse perpetrator-independent/only male—Step 1: *R*^2^ = 0.04, *p* = 0.013; Step 2: *R*^2^ = 0.04, *p* = 0.021; Child abuse perpetrator-independent/only female: Step 1: *R*^2^ = 0.02, *p* = 0.061; Step 2: *R*^2^ = 0.08, *p* < 0.001; CN perpetrator-dependent/gender-non-specific—Step 1: *R*^2^ = 0.1, *p* = 0.044; Step 2: *R*^2^ = 0.03, *p* < 0.001; CN perpetrator-dependent/only male—Step 1: *R*^2^ = 0.01, *p* = 0.366; Step 2: *R*^2^ = 0.01, *p* = 0.547; CN perpetrator-dependent/only female—Step 1: *R*^2^ = 0.02, *p* = 0.11; Step 2: *R*^2^ = 0.09, *p* < 0.001; CPA perpetrator-dependent/gender-non-specific—Step 1: *R*^2^ = 0, *p* = 0.365; Step 2: *R*^2^ = 0.02, *p* = 0.004; CPA perpetrator-dependent/only male—Step 1: *R*^2^ = 0.01, *p* = 0.252; Step 2: *R*^2^ = 0.01, *p* = 0.416; CPA perpetrator-dependent/only female: Step 1: *R*^2^ = 0, *p* = 0.929; Step 2: *R*^2^ = 0.08, *p* < 0.001; CEA perpetrator-dependent/gender-non-specific—Step 1: *R*^2^ = 0.02, *p* = 0.006; Step 2: *R*^2^ = 0.04, *p* < 0.001; CEA perpetrator-dependent/only male: Step 1: *R*^2^ = 0.03, *p* = 0.011; Step 2: *R*^2^ = 0.03, *p* = 0.03; CEA perpetrator-dependent/only female—Step 1: *R*^2^ = 0.02, *p* = 0.057; Step 2: *R*^2^ = 0.09, *p* < 0.001

*p ≤ 0.05, **p ≤ 0.01, ***p ≤ 0.001

### Child abuse and SR-Men

#### Correlation analyses

A significant positive correlation was found between CN and SR-Men parent-non-specifically and specifically. CEA_m_ was positively associated with SR-Men. CPA did not correlate with SR-Men. For further information see Additional file 2: bivariate associations/correlation analyses.

#### Regression analyses

In regression analyses, controlling for BMI and SES, effects of subtypes of child abuse on SR-Men were not significant. Throughout all analyses a higher BMI and a low SES significantly predicted early menarche, except in analyses contrasting all three forms of abuse. See Table 5

**Table 5:** Regression analyses SR-Men—child abuse

Predictor	β	*B (SE)*	95% CI for *B*	
			Lower	Upper
Child abuse perpetrator-independent				
Step1				
CN	0.13	0.14	**−** 0.01	0.53
CPA	0.03	0.17	**−** 0.27	0.41
CEA	0.09	0.15	**−** 0.11	0.48
Step2				
CN	0.10	0.14	**−** 0.08	0.46
CPA	0.02	0.17	**−** 0.28	0.39
CEA	0.08	0.15	**−** 0.13	0.45
BMI	0.20**	0.02	0.02	0.09
Child neglect (CN) perpetrator-dependent				
Step1				
CN_m_	0.12	0.16	**−** 0.08	0.55
CN_f_	0.06	0.17	**−** 0.21	0.44
Step2				
CN_m_	0.12	0.16	**−** 0.08	0.54
CN_f_	0.02	0.17	**−** 0.28	0.37
BMI	0.21**	0.02	0.02	0.09
Step3				
CN_m_	0.09	0.16	**−** 0.14	0.49
CN_f_	0.01	0.17	**−** 0.30	0.35
BMI	0.18*	0.02	0.01	0.09
SES middle	**−** 0.11	0.20	**−** 0.61	0.18
SES high	**−** 0.24*	0.21	**−** 0.88	**−** 0.06
Child physical abuse (CPA) perpetrator-dependent				
Step1				
CPA_m_	**−** 0.02	0.16	**−** 0.36	0.27
CPA_f_	0.11	0.18	**−** 0.10	0.60
Step2				
CPA_m_	**−** 0.02	0.16	**−** 0.34	0.27
CPA_f_	0.08	0.17	**−** 0.16	0.52
BMI	0.21**	0.02	0.02	0.10
Step3				
CPA_m_	**−** 0.05	0.16	**−** 0.42	0.20
CPA_f_	0.09	0.17	**−** 0.13	0.55
BMI	0.17*	0.02	0.01	0.08
SES middle	**−** 0.13	0.2	**−** 0.63	0.15
SES high	**−** 0.28**	0.21	**−** 0.95	**−** 0.14
Child emotional abuse (CEA) perpetrator-dependent				
Step1				
CEA_m_	0.13	0.17	**−** 0.06	0.59
CEA_f_	0.05	0.15	**−** 0.2	0.41
Step2				
CEA_m_	0.11	0.16	**−** 0.10	0.54
CEA_f_	0.03	0.15	**−** 0.24	0.36
BMI	0.20**	0.02	0.02	0.09
Step3				
CEA_m_	0.07	0.17	**−** 0.19	0.47
CEA_f_	0.04	0.15	**−** 0.22	0.37
BMI	0.17*	0.02	0.01	0.08
SES middle	**−** 0.13	0.20	**−** 0.63	0.15
SES high	**−** 0.25*	0.21	**−** 0.90	**−** 0.08

*CN*_*m/f*_ child neglect perpetrated by mother/father, *CPA*_*m/f*_ child physical abuse perpetrated by mother/father, *CEA*_*m/f*_ child emotional abuse perpetrated by mother/father, *SES* socioeconomic status, *BMI* Body-Mass-Index, *β* standardized beta value, *B (SE)* standard errors of *B*, *95% CI for B* 95% confidence interval for *Odds Ratio*

Child abuse perpetrator-independent—Step 1: *R*^2^ = 0.04, *p* = 0.038; Step 2: *R*^2^ = 0.08, *p* = 0.002; CN perpetrator-dependent—Step 1: *R*^2^ = 0.03, *p* = 0.077; Step 2: *R*^2^ = 0.07, *p* = 0.003; Step 3: *R*^2^ = 0.1, *p* = 0.001; CPA perpetrator-dependent—Step 1: *R*^2^ = 0.01, *p* = 0.348; Step 2: *R*^2^ = 0.06, *p* = 0.01; Step 3: *R*^2^ = 0.09, *p* = 0.002; CEA perpetrator-dependent—Step 1: *R*^2^ = 0.03, *p* = 0.071; Step 2: *R*^2^ = 0.07, *p* = 0.004; Step 3: *R*^2^ = 0.1, *p* = 0.001

*p ≤ 0.05, **p ≤ 0.01

## Discussion

In this study, we investigated associations between child abuse and pubertal timing. Our analyses offer new insights implying that neglected and emotionally abused children may enter puberty earlier than non-abused children. Moreover, our study showed markedly distinct effects among girls and boys and, more substantially, variation as a function of the sex of perpetrator. Multivariate Analyses, controlling for BMI and SES, revealed CN_m_ to be associated with early physical pubertal development among girls. Regarding CEA, girls with CEA_m_ and boys with CEA_f_ were more likely to enter puberty early. However, when controlling for BMI, the effect of CEA_m_ on girls’ puberty waned. CPA had no effect on pubertal timing. Concerning menarche, parent-specific and non-specific analyses displayed significantly positive correlations between early menarche and CEA_m,_ CN_m_ and CN parent-non-specifically. However, in regression analyses controlling for BMI and SES, significant effects could not be confirmed.

### Child neglect (CN)

Identifying an association between CN_m_ and early puberty, our outcomes supported our first hypothesis and were in line with Brown et al. [38] and Jorm et al. [39]. Although our sex-specific findings found CN_m_ to be linked to early pubertal timing (concerning physical markers) in girls, an association with early menarche could only be seen in correlation, but not be proven in regression analyses. Recapitulating previous studies on CN and puberty, research has led to quite contradictory propositions. Henrichs et al. [40] and Mendle et al. [41], referring only to physical CN, found CN to be associated with early menarche. Others in contrast observed CN to be linked to late puberty [18]. Yet, comparable to our results on girls’ puberty following CN_m_, father’s absence has been repeatedly shown to be associated with early puberty in girls [42]. Taking on the responsibility of both parents as a single-mother can lead to excessive demand. In fact, mothers’ economic hardship may increase parenting stress [43]. Consequently, CN resulting from not being able to fulfil a child’s needs is conceivable. Studying the proposed interrelation between father’s absence and CN_m_ could be of great interest.

### Child physical abuse (CPA)

In contrast to previous research and to our first hypothesis, we did not find significant associations between CPA and pubertal timing. Several studies have displayed girls with experiences of CPA to start puberty early. Due to generally weak effects of CPA on puberty in literature, we might not have been able to detect effects within our sample. Recruitment in child protection services might strengthen the necessary statistical power.

### Child emotional abuse (CEA)

As there are variations in definition, CEA has still only been insufficiently studied in research so far. Referring to DSM-5 [44], CEA is defined as a form of abuse that implies an intentional verbal or symbolic attempt by a parent or caregiver to scare, humiliate, isolate or ignore a child as a consequence of stress, poor parenting skills, social isolation, lack of available resources or inappropriate expectations of their children [44]. To our knowledge, this was one of the first studies to examine associations between CEA and pubertal timing. An exception was the work by Li et al. [18] showing CEA among other forms of child abuse to be associated with late menarche in girls and late gonadal hair growth in boys. In contrast to these findings but along with our first hypothesis, we found boys with CEA_f_ and girls with CEA_m_ (only in correlation analyses) to start puberty earlier. Specifying CEA as a general threat-related adversity our results resembled findings by Sumner et al. [45] and Colich et al. [46] examining ACE and biological aging. They divided data on ACE into two groups, threat-related adversities, expressing experiences involving harm or threat of harm to the child (e.g. CEA) and deprivation-related adversities, referring to absence of expected inputs from the environment during development (e.g. cognitive and social stimulation). In both studies, threat-related experiences were significantly associated with accelerated puberty, more significantly in cases of no co-occurrence of deprivation. Concerning future research, the idea of distinguishing between threat and deprivation related adversities should be enhanced.

### The role of sex and perpetration

In our study we found sex-specific effects between child abuse and pubertal timing as assumed in our third hypothesis. Our results showed CN_m_ leading to early puberty in girls and CEA_f_ to early puberty in boys. Many studies indicate on the role of sex in understanding psychopathology [47]. Prevalence rates of child abuse vary among boys and girls. Cui et al. [24], for example, observed girls to be more likely than boys to be neglected. Our descriptive data support this finding. Concerning our results on the sex-specific impact of child abuse on pubertal timing, we assume them to be due to gender-specific variations in stress responses. Studies such as by Del Giudice et al. [20] and Bangasser et al. [21] postulated especially females to be more sensitive to stress. Consistently, girls affected by child abuse have been repeatedly observed to start puberty rather early in comparison to boys [14, 15]. In contrast, Negriff et al. [19] detected girls affected by CSA to be associated with early and boys by CN with late pubertal timing. To conclude, girls and boys seem to react differently to certain types of child abuse.

According to our second hypothesis, our analyses were also able to identify variances among sexes concerning the parental perpetration of child abuse. Girls were more likely to start puberty early when being neglected by their mother and boys when emotionally abused by their father. Our analyses suggest that pubertal timing seems under special influence of child abuse perpetration by same-sex parents. This might be due to sex differences in parenting strategies [26] and children’s tendency to imitate same-sex parental behaviour [27].

Concerning all types of child abuse except CEA, our data revealed mothers to perpetrate child abuse more often than fathers. Boys were affected more often by CEA_m_ and girls by CEA_f_. CPA generally occurred more often among boys. Cui et al. [24] showed mothers to perpetrate maltreatment against girls more frequent than fathers, except for CN. In our study, the opposite was the case. However, according to Straus et al. [25] perpetration by mother is as frequent as that by father [25]. A meta-analysis by Endendijk et al. [23] on sex-differentiated parental control displayed prevalence of child abuse to have changed over the past centuries, assumably due to changes in parenting styles and socialisation. Overall, Endendijk et al. [23] revealed differences between parenting of boys versus girls to be quite small. As parenting is a dynamic process between the parents [48], abusive behaviour is most commonly (in about half the cases) exhibited by both parents [25, 49]. Therefore, perpetration is difficult to attribute to one parent.

### The impact of BMI and SES

Verifying our fourth hypothesis early puberty was significantly associated with high BMI (especially in girls) and low SES (only concerning menarche) in our analyses. Among boys, there was a significant negative correlation between BMI and CPA. Girls with high BMI were more likely to enter puberty early. Supporting our findings, Kaplowitz et al. [32] identified obesity, Hoyt et al. [33] higher young adult BMI as an important contributing factor for early pubertal timing in girls. Brix et al. [50], however, found higher childhood BMI to be associated with earlier pubertal timing in boys, too. Concerning SES, Deardorff et al. [31], Braithwaite et al. [51] and James-Todd et al. [30] were in line with our study by showing low SES to be associated with early menarche. In contrast to their conclusions, Zhang et al. [42] and Colich et al. [46] displayed no association of low SES with early menarche. We suppose that contradictory outcomes might be due to challenges in methodology and SES definition.

### Limitations and strengths

Several strengths and limitations should be acknowledged. In contrast to prior research, we considered menarche and physical puberty markers. Measurement of menarche relied on retrospective parent report which is subject to recall or reporting biases. Since some girls had not reached menarche at t1 yet, our data were accumulated with information given at t2. Information on tanner stages, assessed by a current self-report, was less likely to be affected by recall bias. Moreover, since the age of participants ranged between 8 and 14, an age correction of data on the pubertal status was indispensable. In consequence, pubertal status does not reflect the exact age at pubertal onset. Since we focused on a child sample, we were not able to evaluate the association of child abuse with late puberty. The CTS is a valid screening instrument for subtypes of child abuse. Abuse measures correspond to conventional definitions. Nevertheless, these are self-reports of forms of child maltreatment and not cases of child abuse verified by experts. Additionally, the CTS only provided us with data on child abuse having taken place within 1 year prior to data assessment, but not with information about age of onset and duration of child abuse. Contrary to previous studies, we conducted both sex-dependent and parent-specific analyses. Our sample included a relatively small number of children with severe forms of child abuse, which is reflected in small effect sizes. Because of statistical power and small group sizes, we did not differentiate between community and clinical sample. Even though our sample encompassed a high variance of different family environments, we did not recruit families in child protection services. This could be useful for further studies to strengthen statistical power. Contradictory outcomes in research on child abuse and puberty might be due to variances in definition or assessment of child abuse. Therefore, standardized measurement techniques of child abuse should be pursued. In general, meta-analyses and systematic reviews are needed to intensify examinations on pubertal timing, the role of sex and the sex-specific effect of perpetration.

## Conclusions

Our study extended previous work on child abuse and pubertal development by investigating the onset of puberty focussing on children having experienced child abuse and attempting to identify other mediating influences such as BMI and SES. Our results exhibited child sex and identity of perpetrator to play a substantial role on pubertal development concerning physical pubertal markers and onset of menstruation among children with experiences of child abuse, especially CN and CEA.

Girls having suffered from CN perpetrated by their mother and boys having suffered from CEA perpetrated by their father can be identified more easily as being at risk of entering puberty early and as a consequence, developing potential physical and mental health damages associated with early pubertal development, for example depression [10] and asthma [11]. Knowledge of sex- and perpetrator-specific effects of CN and CEA could help clinicians to specify their diagnostic process and to define differential prevention and treatment goals.

Further testing of empirical models with the parameters discussed is needed to determine the direction of the impact: onset of puberty, menstruation, SES, BMI among children who have experienced CEA and CN. Moreover, as suggested in our paper the role of child sex and identity of perpetrator should be considered in future analyses. Explanations why perpetration by females and males influence pubertal development of girls and boys differently are still missing. We suggest future research to place special focus on social mechanisms, parenting styles, as well as possible mother–daughter and father–son linkages concerning the impact of parental childhood abuse, especially CN and CEA, on children’s pubertal development.

### Supplementary Information

**Additional file 1.** Subscale items of parent–child conflict tactics scale (CTS-PC).

**Additional file 2.** Bivariate associations between early puberty/menarche and child abuse.

## Data Availability

The data that support the findings of this study are available from LIFE—Leipzig Research Centre for Civilization Diseases at the University of Leipzig but restrictions apply to the availability of these data, which were used under license for the current study, and so are not publicly available. Data are however available from the authors upon reasonable request and with permission from LIFE—Leipzig Research Centre for Civilization Diseases at the University of Leipzig.

## Abbreviations

ACE: Adverse childhood experiences
CN_m/f_: Child emotional and physical neglect (perpetrated by mother/father)
CEA_m/f_: Child emotional abuse (perpetrated by mother/father)
CPA_m/f_: Child physical abuse (perpetrated by mother/father)
CSA: Child sexual abuse
SR-Pub/Men: Standardized residual variable expressing early puberty/menarche
t1/t2: First/second time of data assessment

## Footnotes

1 Data of sex are referring to biological sex of participants.
